# Single-Cell Profiling of the Immune Atlas of Tumor-Infiltrating Lymphocytes in Endometrial Carcinoma

**DOI:** 10.3390/cancers14174311

**Published:** 2022-09-02

**Authors:** Fang Jiang, Yuhao Jiao, Kun Yang, Mingyi Mao, Mei Yu, Dongyan Cao, Yang Xiang

**Affiliations:** 1 Department of Obstetrics and Gynaecology, Peking Union Medical College Hospital, Chinese Academy of Medical Sciences & Peking Union Medical College, National Clinical Research Centre for Obstetric & Gynaecologic Diseases, Beijing 100730, China; 2 Department of Medicine, Peking Union Medical College Hospital, Chinese Academy of Medical Sciences & Peking Union Medical College, Beijing 100730, China; 3 Department of Dermatology, Beijing Hospital, National Centre of Gerontology, Institute of Geriatric Medicine, Chinese Academy of Medical Sciences, Beijing 100730, China

**Keywords:** tumor-infiltrating lymphocytes, endometrial carcinoma, single-cell RNA sequencing, immune checkpoint molecules, natural killer cell, CD103

## Abstract

**Simple Summary:**

Immunotherapy has a unique potential for improving various types of cancer treatment. However, related studies in endometrial carcinoma are lacking. Further studies on the tumor-infiltrating lymphocyte populations are vital. We firstly mapped the immune atlas of lymphocytes in type I endometrial carcinoma via single-cell RNA sequencing, mass cytometry and flow cytometry analysis. Three transcriptionally distinct NK cell subsets with different antitumor functions were identified. Some CD103+ CD8+ T cells were also defined as tissue-resident memory T cells in endometrial carcinoma. Both our retrospective study and analysis of a public repository suggest the correlation between antitumor function of CD103+ CD8+ T cells and EC prognosis. Certain key molecular mechanisms potentially determining the phenotypes of NK cells and CD103+ CD8+ T cells were highlighted and might serve as novel therapeutic targets.

**Abstract:**

Endometrial carcinoma (EC) is a gynecological malignancy with a high incidence; however, thorough studies on tumor-infiltrating lymphocyte (TIL) populations in EC are lacking. We aimed to map the immune atlas of TILs in type I EC via single-cell RNA sequencing (scRNA-seq), mass cytometry and flow cytometry analysis. We found that natural killer (NK) cells and CD8+ T lymphocytes were the major components of TILs in EC patients. We first identified three transcriptionally distinct NK cell subsets, which are likely to possess diverse anti-tumor functions. Additionally, CD103+ cells substantially contributed to the CD8+ T cell population. The signature gene expression of CD103+ CD8+ T cells indicated the tissue residency, immunological memory, and exhaustion properties of this cell subset, which were defined as tissue-resident memory T cells (T_RM_ cells). Moreover, based on scRNA-seq and mass cytometry analysis, we first identified the intrinsic heterogeneity of CD103+ CD8+ T cells that were thought to have a distinct cytotoxicity, cell adhesion and exhaustion status functions. Collectively, distinct subsets of NK cells were found and might shed light on future investigations. CD103+ CD8+ T cell population may be an important immunotherapeutic target in EC and targeting this cell population with combined immunosuppressive therapy might improve the efficacy of immunotherapy for EC.

## 1. Introduction

Endometrial carcinoma (EC) is the most common gynecological malignancy worldwide [1]. In 2019, EC was the sixth most common cause of death in females in the United States and ranked fourth among cancers in terms of the number of new cases of cancer [2]. Immunotherapy has revolutionized the treatment of tumors, but clinical trials of immune checkpoint inhibitors for EC have comparatively limited sample sizes and varied inclusion criteria. Moreover, current research shows substantial heterogeneity in outcomes among patients with advanced endometrial cancer. The objective response rate after treatment with anti-programmed cell death protein 1 (PD-1) antibodies ranges from 13–56% [3]. Numerous tumor studies have shown that the composition and status of tumor-infiltrating lymphocytes (TILs) are closely related to the treatment response and prognosis of patients with tumors [4]. Since cancers originating in different organs differ in many aspects, extrapolation from one organ to another is not possible. On the other hand, research on the features of the tumor immune microenvironment is as pivotal as research on the characteristics of the tumor itself for identifying an appropriate immunotherapeutic strategy and predicting its clinical outcomes. TILs in different tumors are highly heterogeneous in terms of immune cell composition, marker gene expression and functional characteristics, which may explain the varying efficacies of immunotherapy against different tumors [5].

Regarding TILs in EC, retrospective studies based on immunohistochemistry have shown that the total number of T cells in the tumor epithelium is decreased in advanced and high-risk groups and is an independent prognostic factor for disease-free survival in patients with early-stage cancer [6]. A large number of CD8+ T cells and an elevated number of memory T cells in the TIL population are strongly correlated with a good tumor prognosis and prolonged survival [7]. The number of CD8+ CD103+ TILs in the epithelium is also a relevant factor for the prognosis of high-risk patients with EC [6]. However, recent studies have only analyzed the correlations between the numbers of certain T cells and disease prognosis, and the immunological features and functions of important components of TILs in EC are less well-characterized than those of TILs in other tumors. Hence, we wish to fill this gap by comprehensively analyzing the transcriptomic features and immunophenotypes of TILs in EC. We adopted strategies including flow cytometry, single-cell RNA sequencing (scRNA-seq) and mass cytometry to define the immunological landscape of TILs in EC in an unbiased manner and to further investigate the characteristics of certain significant populations of TILs and their relevance to clinical prognosis and potential as targets for immunotherapy.

## 2. Materials and Methods

### 2.1. Patient Samples

This study was approved by the Ethics Committee of Peking Union Medical College Hospital (PUMCH) (JS-2163). Patients diagnosed with EC with a histopathological type of endometrioid carcinoma between October 2018 and January 2020 at PUMCH were included in the study. All the included patients had a preoperative pathological diagnosis, and their pathology was confirmed after surgery. The control group included patients who underwent total hysterectomy for uterine prolapse. Patients with immune system diseases, infections or other malignancies were excluded. Peripheral blood was obtained before treatment, including chemotherapy, radiotherapy, surgery and immunotherapy. Endometrial or tumor tissue samples were obtained from the patients in both groups immediately after the removal of the uterus. Peripheral blood mononuclear cells (PBMCs) were purified from whole blood with a Ficoll-Paque PLUS (GE Healthcare, Chicago, IL, USA) gradient and cryopreserved prior to analysis. Tumor specimens were prepared as described below. Under sterile conditions, tumors were cut into small pieces and digested in RPMI-1640 medium supplemented with 10% fetal calf serum, 1 mg/mL collagenase type IV (Gibco, Carlsbad, CA, USA), and 30 U/mL DNase I (New England Biolabs, Ipswich, MA, USA). Cells were digested for 30 min at 37 °C at 220 rpm. The cell suspensions were filtered through a 70 μm filter. TILs were enriched by performing 40% and 80% Percoll density gradient centrifugation. Single-cell suspensions were cryopreserved until further analysis.

### 2.2. Antibodies and Flow Cytometry

The following fluorophore-labelled antibodies were used: PE/Cy7-conjugated anti-human CD3 (UCHT1), allophycocyanin (APC)/Cy7-conjugated anti-human CD8 (SK1), APC-conjugated anti-human CD103 (Ber-ACT8), PerCP/Cy5.5-conjugated anti-human CD45RO (UCHL1), fluorescein isothiocyanate (FITC)-conjugated anti-human CD45RA (HI100), APC/Cy7-conjugated anti-human CD69 (FN50), Alexa Fluor (AF) 647-conjugated anti-human CD49a (TS2/7), FITC-conjugated anti-human CD279 (PD-1) (EH12.2H7), PE-conjugated anti-human CD152 (cytotoxic T lymphocyte-associated protein-4, CTLA-4; BNI3), PerCP/Cy5.5-conjugated anti-human CD366 (T cell immunoglobulin and mucin domain-containing 3, Tim-3) (F38-2E2), PE-conjugated anti-human CD197 (Ccr7) (G043H7), PerCP/Cy5.5-conjugated anti-human CD223 (LAG-3) (11C3C65), PE-conjugated anti-human T cell immunoreceptor with Ig and immunoreceptor tyrosine-based inhibition motif (ITIM) (TIGIT; VSTM3) (A15153G) (all from BioLegend, San Diego, CA, USA), and FITC-conjugated anti-human CD45 (HI30; BD Pharmingen™, New York, NY, USA). Propidium iodide solution and 7-AAD (Invitrogen, Carlsbad, CA, USA) were used for live/dead staining according to the manufacturer’s instructions. Stained cells were sorted on an LSRFortessa flow cytometer (BD Biosciences, Franklin Lakes, NJ, USA) or a FACSAria II (BD Biosciences). Data were analyzed with FlowJo software (BD Bioscience, USA).

### 2.3. Mass Cytometry

Mass cytometry was conducted according to the instructions provided by Fluidigm. Metal-conjugated antibodies for mass cytometry (cytometry by time-of-flight, CyTOF) were obtained from Fluidigm and are listed in Table S1. An anti-human Hobit antibody was purchased from BD and labelled with a MaxPar DN3 labelling kit from Fluidigm. The antibodies were all titrated. Single-cell suspensions were first incubated with cisplatin to assess viability and then washed with PBS containing 1% BSA. The cells were then stained with an antibody cocktail for 30 min at room temperature. After washing with PBS containing 1% BSA, the cells were fixed with 1.6% paraformaldehyde overnight. After two washes with PBS containing 1% BSA, the cells were permeabilized with cold methanol. Cells were then incubated overnight at −20 °C and washed twice with PBS containing 1% BSA. The cells were washed and then stained with an iridium DNA intercalator for 20 min at room temperature. After two washes with PBS containing 1% BSA and two washes with ddH_2_O, the cells were resuspended in 1× EQTM Four Element Calibration Beads and analyzed with a CyTOF 1.0 mass cytometer. Eight thousand TILs from five patients with endometrioid carcinoma were analyzed using mass cytometry. An unsupervised clustering analysis was applied using “PhenoGraph”, and dimension reduction was completed via t-distributed stochastic neighbor embedding (t-SNE). The clusters were annotated according to the expression pattern of the markers.

### 2.4. Single-Cell RNA Sequencing

Fresh tumor samples obtained during surgery were immediately processed using enzymatic digestion to isolate single-cell suspensions of TILs, which were then sorted on a FACSAria II. The scRNA-seq approach utilized in this project was conducted according to the Chromium™ Single Cell 3′ v2 protocol provided by 10× Genomics. Approximately 10,000 sorted CD45+ cells were sampled for each experiment. After the initial processing, data cleaning and merging steps, 28,820 cells were analyzed. The Cell Ranger™ analysis pipelines were able to process the raw BCL dataset generated by Illumina. R and Python were the two major programming languages used in this project. Crucial open access packages, including “Seurat (R, version 3.0)” [8,9], “Monocle 3 (R)” [10,11,12], and “CellPhoneDB (python, version 2.0)” [13], were obtained online. Briefly, dying cells, cell doublets and low-quality cells were first removed. The clean data from the three datasets were then merged and transformed. For dimensionality reduction, both the UMAP [14] and t-SNE [15] methods were applied. A built-in graph-based clustering analysis from “Seurat” was used to determine clusters in an unbiased manner. Clusters were annotated according to differentially expressed genes.

### 2.5. RNA-Seq

CD103+ CD8+ T cells and CD103− CD8+ T cells were sorted on a FACSAria II and then stored in TRIzol (Invitrogen) at −80 °C. Total RNA was purified using a SMART-Seq V4 Ultra Low Input RNA kit for Sequencing 480 Rxns (catalog No. 634895). The purified total RNA was amplified according to the smart-seq2 protocol. After construction of the library, a Qubit 2.0 fluorometer was used to quantify the initial concentration. The cDNA library was diluted to 1.5 ng/µL. The insert size was examined with an Agilent 2100 bioanalyzer. Final quantification (effective concentration > 2 nM) was conducted using qRT–PCR if the insert size met the standard to ensure the high quality of the library. Different libraries were pooled according to the effective concentration and target data size for Illumina sequencing, generating 150 bp paired-end reads.

Gene ontology (GO) enrichment analysis of the differentially expressed genes was performed with the clusterProfiler R package, with correction for gene length bias. GO terms with corrected *p* values less than 0.05 were considered significantly enriched with differentially expressed genes.

### 2.6. Immunohistochemistry

Fixed, paraffin-embedded EC tissue blocks from appropriate patients were obtained from the Pathology Library of PUMCH. Sections with a thickness of 4 µm were cut and mounted on amino-propyl-tri-ethoxy-silane-coated slides. The sections were then deparaffinized with xylene and ethanol and rehydrated with water. The sections were incubated with Tris/EDTA buffer with the pH adjusted to ~9.0 in a 60 °C water bath overnight. Sections were then stained with immunohistochemical reagents using the BOND-MAX (Leica^®^, Wetzlar, Germany) automated immunohistochemistry system. All sections were then scanned to produce digital images. CD8 and CD103 double staining was performed using a CK (pan)/p63 immunohistochemical double staining kit (Origene, Rockville, MD, USA, DS-1101). The following antibodies were used: anti-CD103 (Boster, Wuhan, China, 1:200) and anti-CD8 (Cell Marque, Rocklin, CA, USA, C8/144B, 1:30). TILs were quantified using a Nikon Eclipse 80I microscope. The three independent areas with the most abundant lymphocyte infiltration were selected. The numbers of TILs and positively stained lymphocytes were manually counted in each microscopic field with a 200× objective (0.94985 mm^2^).

### 2.7. Statistics

All statistical analyses were performed with GraphPad Prism 7 software (GraphPad, San Diego, CA, USA) and were two-sided. Significance was set to *p* < 0.05. All bar graphs show the means and SEM. Significance is indicated as follows: * *p* < 0.05, ** *p* < 0.01, *** *p* < 0.001 and **** *p* < 0.0001.

## 3. Results

### 3.1. CD8+ T Cells and NK Cells Are Enriched among TILs in EC

**Figure 1:**
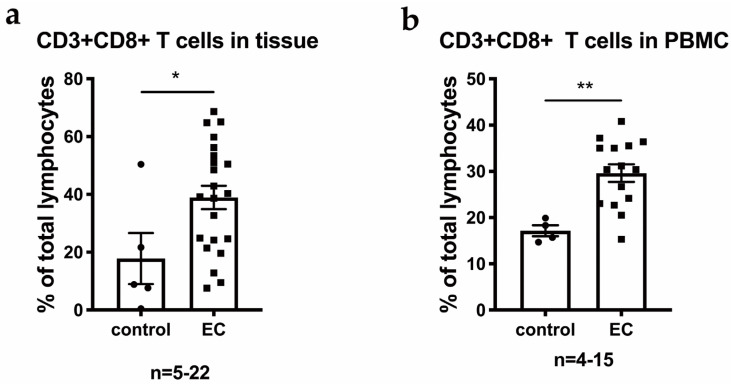
CD8 T cells are enriched in TILs from EC. (**a**) The percentages of CD8 T cells among all lymphocytes in EC tissue, prolapsed uterus (control) and (**b**) PBMCs from the corresponding patients. *p* value compares the indicated group using an unpaired *t*-test (two-tailed). p<0.05 (*), p<0.01 (**).

EC-infiltrating lymphocytes were isolated and analyzed using multiparameter flow cytometry to explore the immunological characteristics of EC (*n* = 22, Table 1). Additionally, lymphocytes in endometrial tissue from patients with pelvic organ prolapse (POP) were collected and analyzed as controls. Lymphocytes were enriched in the EC tissue, but low frequencies of lymphocytes were detected in the prolapsed endometrium (Figure 1a). The average frequencies of CD8+ T cells in both EC TILs and PBMCs from patients with EC were significantly higher than those from control patients (Figure 1b). Based on the immune cell composition of TILs in EC, local inflammation and an expansion of cytotoxic immune cells were identified. Therefore, we further performed scRNAseq and CyToF experiments to explore the properties of TILs in EC.

### 3.2. Single-Cell Transcriptomic Analysis and Mass Cytometry Analysis Comprehensively Revealed the Cellular Composition of TILs

**Figure 2:**
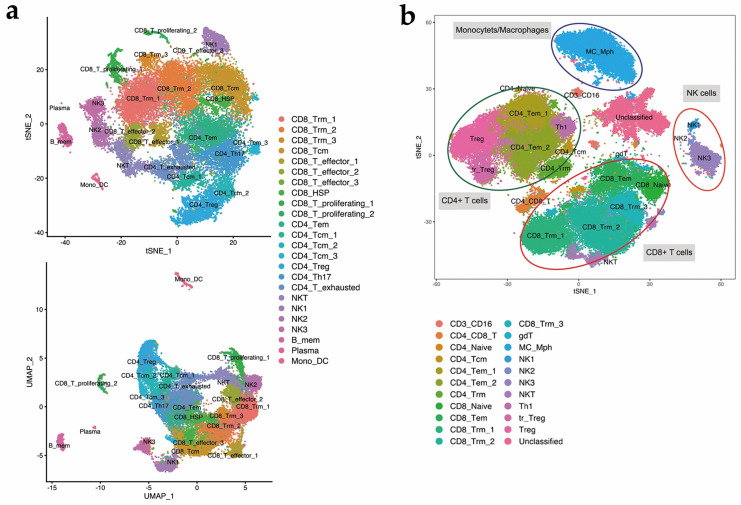
Single-cell transcriptomic and mass cytometry analysis of EC TILs. (**a**) tSNE analysis and UMAP methods were applied for the nonlinear dimension reduction analysis. Clusters were determined with the built-in graph-based method. Each cluster was annotated with a cell type according to marker gene expression; (**b**) Eight thousand TILs from 5 patients with type I EC were analyzed using mass cytometry. Unsupervised clustering analysis was applied using “PhenoGraph”, and dimension reduction was completed using tSNE. Clusters were annotated according to the expression pattern of marker proteins. Three NK cell subsets and 3 TRM subsets were identified, consistent with the findings from scRNA-seq.

T cells and NK cells comprised the major proportions of EC TILs (Figure 2). We isolated pan-CD45+ lymphocytes from the TIL populations of three patients with EC using flow cytometry and performed single-cell 3′ RNA sequencing (scRNA-seq) to analyze these TILs and obtain a deeper understanding of the biology of TILs in EC, including CD8+ T cells. The total numbers of cells estimated to be present in the three scRNA-seq samples were 10,517, 9410 and 8893. The median numbers of genes per cell in the three scRNA-seq samples were 397, 639 and 749. A dimension reduction analysis by t-SNE uniform manifold approximation and projection (UMAP) (Figure 2a) and unsupervised clustering analysis were applied to merged data from the three independent scRNA-seq experiments. Twenty-four clusters were identified and annotated with the appropriate cell types according to transcriptional features. These clusters mainly included distinct NK cell clusters and T cell clusters. The heatmap of the top ten differentially expressed genes in each cluster is shown in Figure S1a.

Regarding NK cells (Figure 2a), we identified three subsets that were separated by dimensional reduction analysis. Two subsets were equal to the canonical peripheral human NK cell subsets, which are CD16+ CD56^dim^ NK cells and CD16− CD56^bright^ NK cells and were labeled “NK1” and “NK3”, respectively. Another noncanonical population was labeled “NK2”. As shown in Figure 2a, 17 clusters of T lymphocytes were identified, among which 10 clusters were classified as CD8+ T cells, and the remaining 7 clusters were considered CD4+ T cells. Some canonical T cell subsets, including CD8+ effector T cells, CD8+ memory T cells, and CD4+ central memory T cells, showed heterogeneity and were clustered into several subsets. When focusing on CD8+ T cells, one major subset was not classified as either TEM cells (defined as SELL− CCR7− S1PR1+ KLF2+ CD69− T cells) or TCM cells (defined as SELL+ CCR7+ KLF2+ S1PR1+ CD69− T cells) [16] due to their unique expression of PRDM1 (B lymphocyte-induced maturation protein (Blimp)) 1 and ZNF683 (Hobit). This subpopulation of T cells is a well-documented subset found in other types of tumors and is classified as tissue-resident memory T (T_RM_) cells.

**Figure 3:**
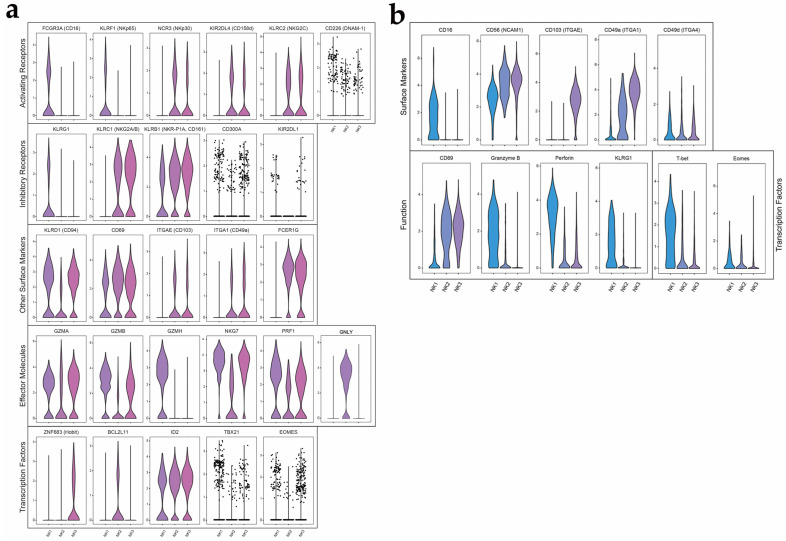
NK cell subset by single-cell RNA sequencing and mass cytometry: (**a**) Expression levels of selected markers in NK cell subsets (scRNA-seq). The expression levels of groups of genes that represent activating receptors, inhibitory receptors, effector molecules, transcription factors and other surface markers are shown; (**b**) The expression levels of certain surface markers, NK cell function-related markers and transcription factors by mass cytometry.

scRNA-seq is an unbiased method for clustering cells and identifying novel immune subsets. To confirm the findings from scRNAseq at the protein level, we harvested TILs from another five patients with type I EC (confirmed by pathology) and performed a mass cytometry analysis to consolidate the findings from the expression data. We identified 22 clusters according to differentially expressed surface markers (Figure 2b). A heatmap of the expression levels of all the surface and intracellular markers is shown in Figure S1b. The four major “islands” were identified as CD4+ T cells, CD8+ T cells, NK cells and monocytes/macrophages (“MC_Mph”). Due to the lack of certain markers, a cluster of cells could not be classified. According to the unsupervised clustering analysis, subsets of NK cells and CD103+ CD8+ T_RM_ cells matched the findings from scRNA-seq. The results from mass cytometry validated our findings for the three NK cell subsets (Figure 3b). For the CD8+ T cell subsets, the results from mass cytometry were heterogeneous, but the major component was still T_RM_ cells expressing CD103, which were also divided into three subsets in accordance with the scRNA-seq results.

### 3.3. Identification of a Novel NK Cell Subset by Single-Cell RNA Sequencing and Mass Cytometry

Human NK cells can circulate in the periphery and reside in tissues. Additionally, it is commonly accepted that these NK cells, regardless of their circulation status, consist of two major subsets, CD16+ CD56^dim^ and CD16+ CD56^bright^, defined by the expression level of CD56 and CD16. However, according to our scRNAseq and CyTOF data, a third subset of NK cells was identified in TILs from EC. We further analyzed the three subsets of NK cells (Figure 3a). For effector molecules, we found that NK1 cells and NK3 cells expressed high levels of Granulysin (GNLY), Granzyme B (GZMB) and Granzyme H (GZMH), and the Achemokine CCL4 compared to NK2 cells. Granzyme A (GZMA) and Granzyme B (GZMB) levels were low in the NK2 subset, indicating that this subset might have the weakest cytotoxicity [17,18]. NK2 cells, however, showed an increased expression of glucocorticoid-induced tumor necrosis factor receptor (TNFR)-related protein (GITR) and the lectin-like inhibitory receptor NKG2A (KLRC1) compared to NK1 cells. Furthermore, the expressions of the activating receptors, NKp65 (KLRF1) and DNAM-1 (CD226), and the inhibitory receptor KLRG1 were elevated in the NK1 subset. The activating receptors, NKp30 (NCR3), CD158d (KIR2DL4) and NKG2C (KLRC2), and the inhibitory receptor KLRC1 (encoding for NKG2A and NKG2B) and CD161 (KLRB1) were elevated in the NK3 subset. The novel NK2 subset showed an intermediate expression of all the markers described above. However, based on the scRNAseq results, NK2 cells were found to express low levels of CD94 (KLRD1), whereas the other two subsets had high expressions. For effector molecules, transcription factors and other markers, expression of integrins such as CD103 (ITGAE) and CD49a (ITGA1), and the transcription factor Hobit (ZNF683), which is considered to be related to tissue-residency markers, were high in the NK3 subset [19,20]. These results imply that NK3 cells are likely to have tissue-resident properties. The transcriptional feature of the three NK cell subsets matched the expression level of markers encoded by the respective genes detected by flow cytometry.

The results from mass cytometry also validated our findings in the three NK cells subsets (Figure 3b). There was an increase in CD103 and CD49a levels in NK3 cells, compared to the other two subsets. Granzyme B, perforin and T-bet levels were highest in NK1 cells, indicating that this subset could be the major contributor to cytotoxicity in EC.

### 3.4. CD103+ CD8+ T Cells Are the Main Component of CD8+ T Cells

Among immune cells exerting anti-tumor function, CD8+ T cells were a critical cell subset. We found CD103+ CD8+ T cells are the main component of CD8+ T cells. Flow cytometry and immunochemical staining were conducted to further investigate CD103+ CD8+ T cells. Bulk RNA-seq was performed to analyze CD103+ CD8+ and CD103− CD8+ T cells and to identify the core transcriptional characteristics of CD8+ CD103+ T_RM_ cells.

**Figure 4:**
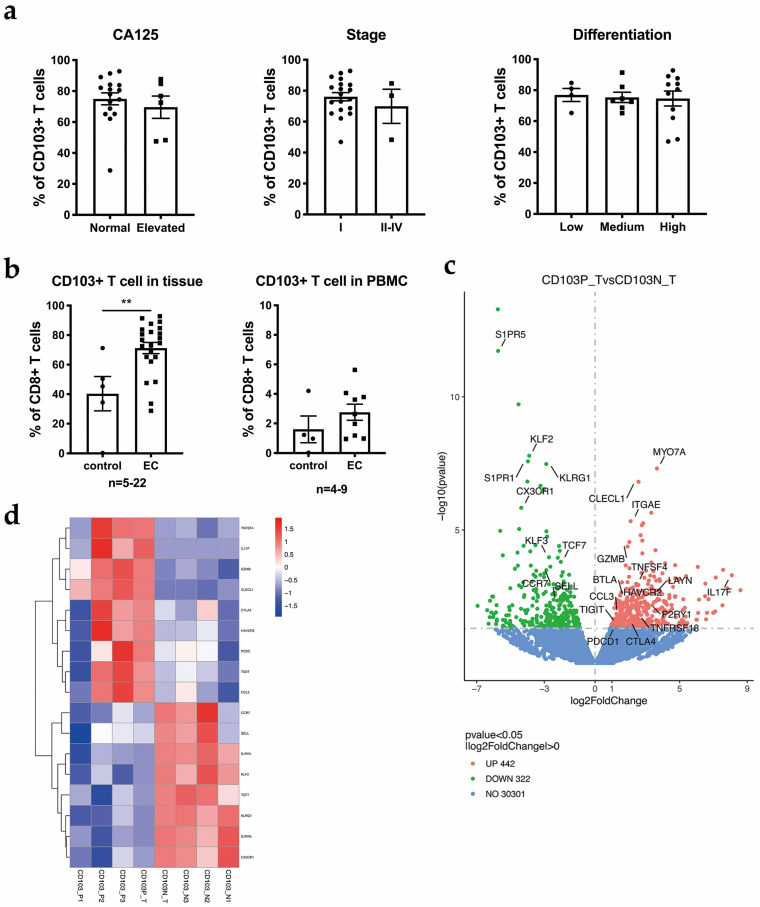
CD103+ CD8+ T cells are the main components of CD8+ T cells: (**a**) The relationship between the abundance of CD103+ CD8+ T cells and clinical features, including CA125 levels, tumor stage and tumor differentiation. (**b**) In patients with EC, the percentage of CD103+ CD8+ T cells among all CD8+ T cells was significantly increased in TILs compared to that in the POP controls. However, the percentage of CD103+ CD8+ T cells among all CD8+ T cells was not significantly increased in PBMCs from patients with EC compared to PBMCs from POP control. (**c**) The volcano plot displays significantly differentially expressed genes between CD103+ (CD103P) and CD103− (CD103N) in flow-sorted CD8+ T cells from *n* = 3 independent patients. Unadjusted p values are shown. Upregulated genes in CD103+ CD8+ T cells are marked in red, and downregulated genes are marked in green. (**d**) A heatmap is shown comparing gene signatures derived from the bulk RNA-seq analysis (CD103+ versus CD103−) of three individuals. *p* value compares the indicated group using an unpaired t-test (two-tailed). p<0.01 (**).

We observed that an average of 70% of the CD8+ T cells were positive for CD103 in EC. Regardless of whether the preoperative CA125 level was elevated, tumor differentiation and clinical stage did not alter the proportion of CD103+ cells in the CD8+ T cell population (Figure 4a). The frequency of CD103+ CD8+ T_RM_ cells was significantly increased in EC tissue compared to POP endometrium (Figure 4b).

Using bulk RNA-seq, we identified a large number of transcripts that were differentially expressed in CD103+ CD8+ T cells compared to CD103− CD8+ T cells. We obtained a set of 442 upregulated genes and 322 downregulated genes in CD103+ CD8+ T cells compared to CD103− CD8+ T cells (Figure 4c and Figure S3). We observed a significantly lower expression of genes encoding tissue egress molecules, such as S1PR1 (encoding the G protein-coupled receptor S1P1), KLF2 (encoding the transcription factor Krüppel-like factor (KLF) 2), and Tcf7 (encoding the transcription factor TCF1), in CD103+ CD8+ T cells. The binding of S1PR1 to its ligand (S1P) mediates the egress of T cells from tissues, and the downregulation of S1PR1 expression is required for T cell retention [21]. The downregulation of KLF2 expression was shown to result in a reduced expression of S1PR1 [21]. Moreover, markers of TCM cells, such as Ccr7 and SELL, were expressed at significantly lower levels in CD103+ CD8+ T cells than in CD103− CD8+ T cells. All the data shown above indicate that the CD103+ CD8+ T cells from the TIL population in EC have strong tissue-resident phenotypes and are classified as T_RM_ cells.

**Figure 5:**
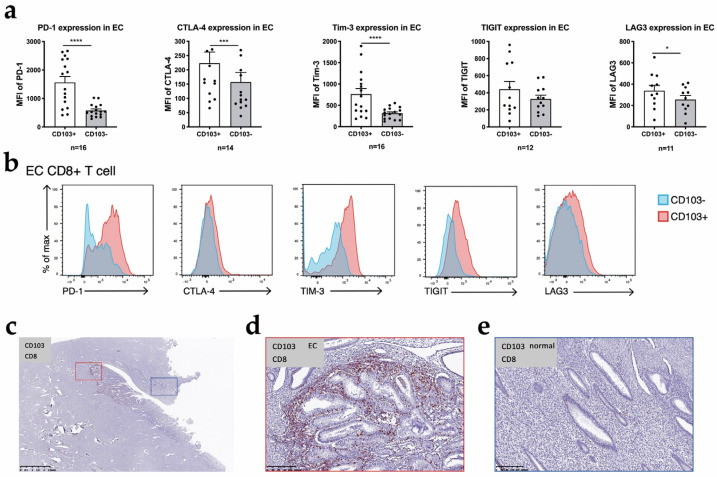
The expression levels of immune checkpoints in T_RM_ cell: (**a**) The expression levels of PD-1, CTLA4, Tim-3 and LAG3 were significantly increased in CD103+ CD8+ T cells; (**b**) One sample of the expression of PD-1, CTLA4, Tim-3 and LAG3 between CD103+ CD8+ T cells and CD103− CD8+ T cells. (**c**) The distribution of CD103+ CD8+ cells detected using double immunohistochemical staining in a whole-slide red box: EC, blue box: normal. Partial magnification of (**d**) EC tumor tissue and (**e**) Adjacent normal endometrium. CD8+: red; CD103+: brown. *p* value compares the indicated group using an unpaired *t*-test (two-tailed). p<0.05 (*), p<0.001 (***), p<0.0001 (****).

The expression of immune checkpoint molecules in CD8+ T cells is very important for determining the employed immunotherapeutic strategies. At the transcriptional level, the bulk RNA-seq analysis comparing CD103+ CD8+ T cells with CD103− CD8+ T cells showed that the expression of multiple genes encoding immune checkpoint molecules was increased in the CD103+ population, including PDCD1 (which encodes PD-1), CTLA4 (which encodes CTLA-4), HAVCR2 (which encodes T cell immunoglobulin and mucin domain-containing 3 (TIM-3)), TIGIT (which encodes TIGIT) and LAG-3 (which encodes lymphocyte-activation gene 3) (Figure 4d). These results agreed with our flow cytometry analysis, which showed that compared to CD103− CD8+ T cells, CD103+ CD8+ T cells exhibited the significantly increased expression of a series of immune checkpoints, including PD-1, CTLA4, Tim-3 and LAG3, indicating that this population was pre-exhausted (Figure 5a,b). TIGIT expression showed the same trend; however, the difference was not significant in flow cytometry analysis.

Based on bulk RNA-seq results, the expression of several transcripts encoding products related to the cytotoxic function of CD8+ T cells, including GZMB, CCL3, FKBP1A, IL-17F and CLECL1, was also significantly upregulated in T_RM_ cells compared with CD103− CD8+ T cells. These transcriptional features of T_RM_ cells in the EC TIL population revealed that T_RM_ cells exhibited a strong potential for cytotoxicity, even with a high expression of a spectrum of immune checkpoint molecules.

After using a GO enrichment analysis of the differentially expressed genes, we found that the upregulated genes are involved in the negative regulation of immune response and immune system process pathways, suggesting that this population was pre-exhausted. (Figure S2) Overall, CD103+ CD8+ T cells show tissue residency and a high expression of molecules linked to cytotoxicity and immune checkpoints. According to the results of immunohistochemical staining, CD103+ T cells were distributed on both sides of the EC tissue. We also used double immunohistochemical staining to show the distribution of CD103+ CD8+ T cells in the tumor tissue and adjacent normal endometrium from an individual patient (Figure 5b).

### 3.5. Heterogeneity Exists within the T_RM_ Cell Subsets in EC

**Figure 6:**
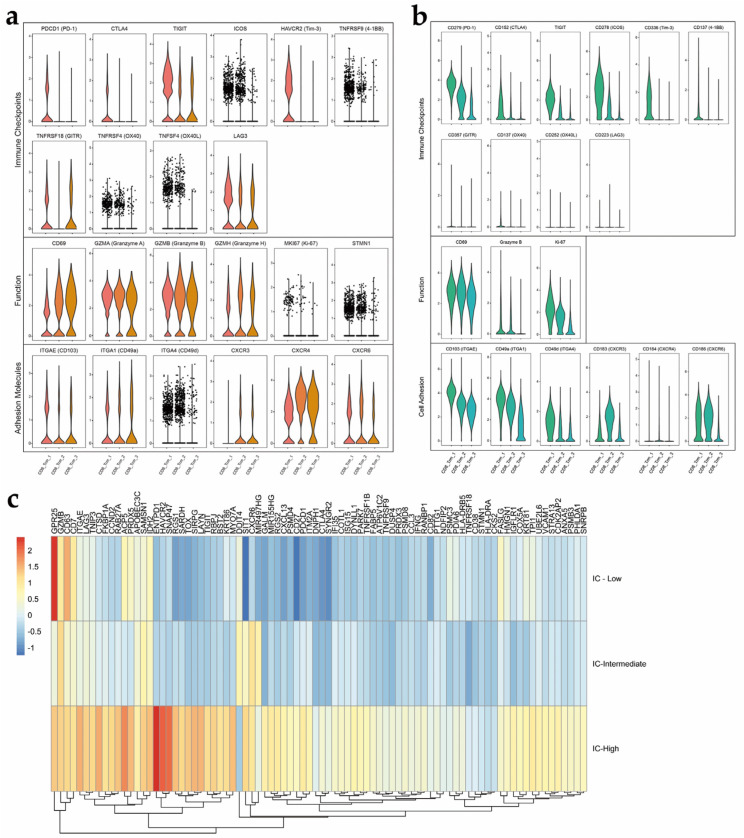
T_RM_ cell subsets in EC show heterogeneity. (**a**) Expression levels of selected markers in T_RM_ cell subsets (scRNA-seq); (**b**) Expression levels of selected markers in T_RM_ cell subsets (mass cytometry). Expression levels of the group of genes that represent immune checkpoint- and cell function (cytotoxicity)-related molecules and adhesion molecules shown in (**a**,**b**); (**c**) Heatmaps comparing genes in TRM subsets. TRM cells were divided into three subsets according to immune checkpoint molecule expression.

According to previous publications, T_RM_ cells are commonly accepted to be memory T cells with tissue residency and characterized by the expression of the key transcription factors ZNF683 and PRDM1 and a range of integrins with tissue specificity [16]. Hence, clusters that were identified in an unbiased manner by either scRNA-seq or mass cytometry with these features were defined as T_RM_ cells in EC. Strikingly, we identified three distinct T_RM_ cell subsets at both the transcript and protein levels (Figure 6a,b).

Among the three T_RM_ cell subsets, the scRNA-seq and mass cytometry results show consistent findings regardless of whether the grouping was based on differentially expressed mRNAs or the markers selected for mass cytometry, including genes/proteins associated with transcription factors, adhesion molecules, cytokine production and effector proteins (Figure 6a,b). Common features include the high expression of the activation marker CD69, which inhibits cell egress from a tissue, at both the transcriptional and protein levels. The mRNA expression of integrins, including CD103, CD49a and CD49d, did not differ significantly, as shown by scRNA-seq. The largest difference was observed in immune checkpoint molecule expression, which led us to name group 3 “IC-high” because it was the major subset that co-expressed multiple immune checkpoint molecules, whereas the other two subsets had low levels of immune checkpoint molecule expression (Figure 6c). Another major finding was that the “IC-high” subset also displayed the highest expression of granzymes. Based on this finding, the “IC-high” subset, a distinct subset of CD103+ CD8+ T_RM_ cells, might have distinct immune functions in such scenarios and requires further study. The “IC-low” subset exhibited the lowest expression of immune checkpoint and cytotoxicity-related molecules at both the transcript and protein levels compared to the other two subsets. The three subsets also differed in the expression of other functional molecules. The “IC-low” and “IC-intermediate” subsets expressed a homolog of Blimp1 found in T cells that directly represses S1PR1 and KLF2, two tissue egress-promoting genes. According to the scRNA-seq data, cell proliferation-related genes such as MKI67 and STMN1 also seemed to be expressed at high levels in the “IC-high” group. At the protein level, Ki-67, which is a marker of cell proliferation, was also detected at higher levels in the “IC-high” cluster than in the other clusters using mass cytometry. In the mass cytometry data, a trend was observed that the expression of three integrins gradually decreased from the “IC-high” to “IC-low” cluster. The “IC-high” and “IC-intermediate” clusters expressed higher levels of CD49a, which is the integrin α1 subunit of very late antigen-1 (VLA-1). In the “IC-intermediate” cluster, we identified a mass cytometry marker that differentiated this subset from the other two subsets: CXCR3. Generally, the “IC-high” cluster seemed to have the strongest cytotoxicity and proliferative features but the highest expression of immune checkpoint molecules. The “IC-intermediate” cluster distinctly expressed CXCR3; however, its biological meaning must be addressed in future experiments. Compared to the other two clusters, the “IC-low” cluster seemed to have the lowest expression of immune checkpoints and cytotoxicity-related molecules at both the transcript and protein levels.

### 3.6. CD103+ CD8+ T Cells Might Be Associated with the Survival in Patients with EC

**Figure 7:**
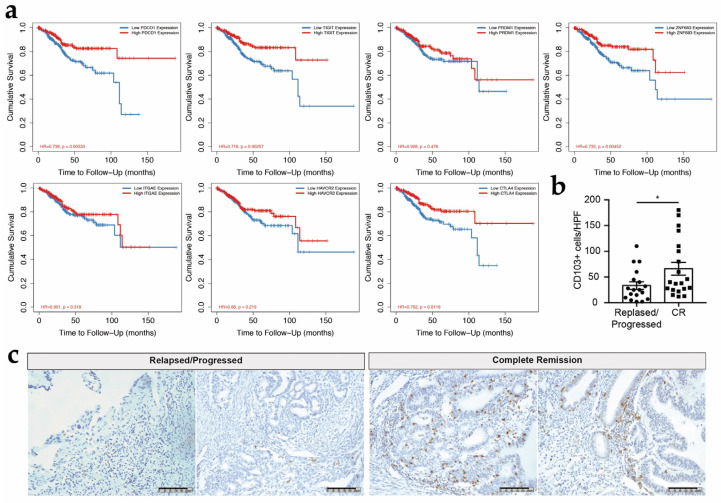
CD103+ CD8+ T cells are associated with a better prognosis for patients with EC. (**a**) Survival analysis shows that high expressions of ZNF683, PRDM1, ITGAE, PDCD1, TIGIT, and CTLA-4 correlate with an improved prognosis. (**b**) The CD103+ cell density is correlated with patient prognosis. CR: complete remission. (**c**) Representative images of immunohistochemical staining of EC tissues from patients who had achieved complete remission for 5 years or relapsed. The data are presented as the means ± SEM from 18 to 20 individuals per group. The p value was obtained from the comparison of the indicated groups using an unpaired *t* test (two-tailed). p<0.05 (*).

We used retrospective prognostic data from the public repository Tumor Immune Estimation Resource (TIMER 2.0) to analyze immune cell infiltration and gene expression and to understand the importance of CD103+ CD8+ T_RM_ cells in the prognosis of EC. The database contains multiple cancer types and corresponding immunological characteristics and clinical outcomes. We chose the cancer type “uterine corpus endometrial carcinoma (UCEC)”, which includes 545 patients, 87 of whom died. The *CD8A* expression level exhibited good correlations with the expression levels of the T_RM_ cell-related key transcription factors *ZNF683* but not *PRDM1*, indicating that the CD8+ T cells in the TIL population contained a significant proportion of T_RM_ cells. Kaplan–Meier survival results show that other factors, including age, clinical stage, and a high expression of ZNF683 were associated with prolonged overall survival (Figure 7a). Regarding the expression of multiple immune checkpoints in TILs, high expressions of PDCD1, TIGIT, and CTLA-4 were associated with prolonged survival.

By comparing the results of immunohistochemical staining from the patients who had achieved complete remission (CR) for 5 years (*n* = 20) with those who relapsed or had progressive disease (*n* = 18), the CD103+ cell density in the relapsed group was significantly lower than that in the CR group (8.3/high-power field (HPF) versus 15.8/HPF, respectively) (Figure 7b,c). Thus, CD103+ CD8+ T cells may predict the survival of patients with EC, which may be related to the high expression of immune checkpoints and suggested that CD103+ CD8+ T cells have antitumor immune functions.

## 4. Discussion

In this study, we used scRNA-seq and mass cytometry to define the immune landscape of TILs in EC in an unbiased manner. The majority of the infiltrated lymphocytes were cytotoxic lymphocytes, including cytotoxic T cells and NK cells, and were consistent with previously published studies.

NK cells, as an important component of the TILs, were first identified by their heterogeneity. Three phenotypically distinct subsets were identified by either mass cytometry or scRNAseq. NK1 cells were likely to be circulatory NK cells that had the strongest cytotoxicity. NK3 cells were shown to have strong features of tissue residency, such as the expression of multiple integrin and the key transcription factor, Hobit, which was proven to be the universal determinator of tissue-resident lymphocytes [16,22]. The novel NK2 cells had a high expression of both CD56 and CD16, showing generally intermediate expression levels of inhibitory and activating receptors, and the weakest expression of effector molecules such as granzymes and perforin, as shown by all the three methods. The results indicate that NK2 cells might be in the phenotypically intermediate stage of the maturation trajectory from NK1 cells to NK3 cells. However, this hypothesis does not agree with the pseudotime analysis, which suggests that NK2 cells might have a distinct origin from NK1 cells and NK3 cells. Hence, to further understand the biology of NK2 cells, more ex vivo and in vitro experiments need to be conducted.

As for T cells, for the first time, we identified a subgroup of CD8+ T cells in EC that express high levels of ZNF683 and PRDM1, which are key transcription factors that guide the tissue residency of lymphocytes and contribute to the process of immunological memory. The high expression of the cell memory marker CD45RO integrins, including CD103 and CD49a, and CD69 identified using flow cytometry, highlight the specific role of this CD8+ T cell subgroup in immunological memory.

These findings indicate the presence of T_RM_ cells in EC. Our in-situ data indicate that T_RM_ cells exert a faster and more powerful killing effect than T_EFF_ cells. They also assist in the aggregation of other immune cells to help quickly clear pathogens or cancer cells. In recent years, T_RM_ cells have been recognized as important functional cells in the TIL population. In some solid tumors, such as breast cancer [23], bladder cancer [24], and non-small cell lung cancer [25], an increase in the number of CD103+ CD8+ T_RM_ cells corresponds to prolonged disease-free survival and overall survival. Although a previous analysis showed that the number of CD103+ CD8+ T cells is associated with the EC prognosis, a further analysis of the phenotypic and transcriptional characteristics of these cells has not been reported. For the first time, we conducted a comprehensive analysis of these cells at the protein and mRNA levels. Based on our results, CD103+ CD8+ TILs display transcriptomic and phenotypic signatures characteristic of T_RM_ cells and might contain heterogeneous subpopulations.

We found that the expression of several transcripts encoding proteins related to the cytotoxic function of CD8+ T cells, including *GZMB*, *CCL3, FKBP1A, IL-17F* and *CLECL1,* was significantly upregulated in T_RM_ cells compared to CD103− CD8+ T cells.

We also utilized a public repository to retrospectively evaluate the relationship between the infiltration of CD103+ CD8+ T_RM_ cells and clinical outcomes. The data show an improved prognosis for patients with higher expression levels of key T_RM_ cell-related transcription factors and surface markers.

The immune checkpoint molecules expressed on T_RM_ cells are reported to vary according to tumor type. In lung cancer, PD-1 and Tim-3 are co-expressed on CD103+ CD8+ T_RM_ cells [25]. In melanoma, PD-1 is co-expressed with LAG-3 on CD103+ CD8+ T_RM_ cells [26]. Only PD-1 is expressed at high levels in CD103+ CD8+ T_RM_ cells in ovarian cancer, and the expressions of CTLA-4, Tim-3 and LAG-3 are negligible [27]. A GO enrichment analysis of differentially expressed genes showed that the significantly upregulated genes were involved in the negative regulation of immune response and immune system process pathways, suggesting that this population was pre-exhausted. Determining the expression of these immune checkpoint proteins might provide insights into choosing an appropriate immune checkpoint blockade (ICB) strategy. The expression profile of immune checkpoint molecules on T_RM_ cells in EC has not yet been reported.

As an important type of immunotherapy, ICB is an important strategy to prevent the exhaustion of cytotoxic T cells and enhance antitumor effects [28]. Monoclonal antibodies against PD-1 and CTLA-4 are among the most widely applied agents in clinical practice [28]. However, their application in EC has limited efficacy, as reported by the KEYNOTE-028 study [3]. The choice of a proper checkpoint to block may be challenging in the clinic. Surface molecules containing an ITIM domain as an intracellular domain are considered to exert an inhibitory effect on cytotoxic T cells upon activation [29]. Therefore, various molecules, including PD-1, CTLA-4, Tim-3, LAG-3, TIGIT, and OX40, are potential targets of ICB. Strikingly, we detected the expression of multiple immune checkpoint molecules, including PD-1, LAG-3, Tim-3, and TIGIT, on CD8+ T_RM_ cells but not by other subsets of CD8+ T cells. However, CTLA-4 expression was not detected using either flow cytometry or scRNA-seq. However, another intriguing finding from the survival analysis is that a higher expression of immune checkpoint molecules correlates with prolonged survival. As T_RM_ cells also displayed an increased expression of cytotoxicity-related molecules, we inferred that this population was the major subset contributing to the antitumor effect; however, T_RM_ cells exhibit a stronger exhaustion phenotype than other tumor-infiltrating T lymphocytes. Reversing exhaustion and enhancing the cytotoxicity of this subset might be key approaches to explore in the future. Among CD103+ CD8+ T cells, cells with high expression of PD-1 also exhibited increased expression levels of CTLA-4, Tim-3, LAG-3 and TIGIT, which highlighted the synergetic inhibitory function of different immune checkpoint proteins and the likelihood of co-inhibition via multiple ligands of immune checkpoints. Hence, due to the overlapping expressions of immune checkpoint molecules, which might explain why single-agent anti-PD-1 monoclonal antibody treatment had limited efficacy, the combination of different ICB therapies might enhance the antitumor effect.

Another novel and critical finding of our study was the heterogeneity of CD8+ T_RM_ cells. The unsupervised clustering analyses based on differentially expressed genes (scRNA-seq) or proteins (mass cytometry) produced similar findings, which show that CD8+ T_RM_ cells were further divided into three distinct subsets. The co-expression of multiple immune checkpoint molecules was mainly observed in one of the subsets, whereas the other two subsets displayed low expressions of immune checkpoint molecules but still expressed cytotoxicity-related molecules at high levels. Hence, we speculate that T_RM_ cells with high co-expressions of multiple immune checkpoint molecules might be the major cytotoxic components acting against tumors. Furthermore, unleashing the power of T_RM_ cells by blocking immune checkpoints might enhance the efficacy of tumor clearance. The most important thing is that once the key driver that skews T_RM_ cells toward immune checkpoint molecule expression is identified in future studies, the corresponding therapeutic strategy can also be clarified.

This study has some limitations. The sample size is not large enough, and the study is descriptive in nature. However, these results have not yet been reported before, and we hope to provide guidance and a reference for future research.

## 5. Conclusions

Based on a thorough and comprehensive immunological mapping of the tumor-infiltrating lymphocytes in EC, we found an accumulation of cytotoxic immune cells, including NK cells and cytotoxic CD8+ T cells. We identified three distinct NK cells subsets. These NK cells subsets were found to show differed patterns of transcriptomes and might possess various anti-tumor effects in the tumor microenvironment. Targeting key molecules such as ZNF683 and other pro-apoptotic proteins might enlighten us on novel NK cell therapies.

Our study provided the first confirmation that the CD103+ CD8+ T cell population among the TILs in EC constitutes T_RM_ cells, with evidence at both the transcriptional and translational levels. Compared with CD103− CD8+ T cells, CD103+ CD8+ T cells exhibited an upregulated expression of various immune checkpoint molecules and enhanced cytotoxic capability. In addition, this population of cells was highly heterogeneous. Collectively, these findings suggested that this population of cells may be an important immunotherapeutic target in endometrial cancer. Combined immunosuppressive therapy targeting this population of cells could improve the efficacy of immunotherapy for EC.

## Figures and Tables

**Table 1:** Characteristics of the patients enrolled for flow cytometry.

Characteristics	Category	*n* (%)
Number of patients	–	22 (100%)
Age (years old)	<45	4 (18.18%)
	≥45	18 (81.82%)
FIGO stage 2009	Ia	14 (63.64%)
	Ib	4 (18.18%)
	II	2 (9.09%)
	III	1 (4.55%)
	IV	1 (4.55%)
Histology type	Endometrioid adenocarcinoma	22 (100%)
Tumor differentiation	Well	10 (45.45%)
	Well–moderate	4 (18.18%)
	Moderate	6 (27.27%)
	Low	1 (4.55%)
	Dedifferentiated	1 (4.55%)
CA125	<35 U/mL	16 (72.73%)
	>35 U/mL	6 (27.27%)

## Data Availability

The data presented in this study are available in this article.

## Supplementary Materials

The following supporting information can be downloaded at: https://www.mdpi.com/article/10.3390/cancers14174311/s1, Figure S1: Heatmaps of differentially expressed genes; Figure S2: Results of the GO enrichment analysis upregulated pathways and downregulated pathways; Figure S3: (a) Histogram and (b) statistic MFI of CD103 expression level in CD8+ T cells; Table S1: Antibodies Used for Mass Cytometry.
