# Supplementary material for: Single-Cell Profiling of the Immune Atlas of Tumor-Infiltrating Lymphocytes in Endometrial Carcinoma
Source: Cancers (Basel). 2022 Sep 2;14(17):4311. doi: 10.3390/cancers14174311 (PMC9455014; doi:10.3390/cancers14174311)
Supplement: Supplementary file 1 [file cancers-14-04311-s001.zip › Supplement figure captions_2022_4_28 1.pdf]

Supplemental Figure Captions

Figure S1 Heatmaps of differentially expressed genes

(A) Differentially expressed genes (transformed across the dataset) in each cluster of cells were analyzed. The top 10 genes with the highest variance were plotted. Expression levels of commonly applied immunological markers are shown in a tSNE plot. **(B)**We used CD3D, CD4, CD8A, IL7R and FoxP3 to identify CD4+ and CD8+ T cells. SELL (CD62L) and Ccr7 were used as markers of the memory phenotype of T cells. NKG7 and FCGR3A (CD16) were used to identify NK cells. CD79A is a marker for B cells. CD14, CLEC10A and FCER1A indicated the phenotypes of monocytes and DCs.

Figure S2 Results of the GO enrichment analysis upregulated pathways and downregulated pathways (BP indicates biological progress, CC indicates cellular component, and MF indicates molecular function).

Figure S3 (a) Histogram and (b) statistic MFI of CD103 expression level in CD8+ T cells. CD103 expression level of CD8+ T cells in patients with EC was increased compared to that in the POP controls. In PBMCs, CD103 expression among all CD8+ T cells showed no difference between patients with EC and POP control.
