# Supplementary material for: Single-Cell Profiling of the Immune Atlas of Tumor-Infiltrating Lymphocytes in Endometrial Carcinoma
Source: Cancers (Basel). 2022 Sep 2;14(17):4311. doi: 10.3390/cancers14174311 (PMC9455014; doi:10.3390/cancers14174311)
Supplement: Supplementary file 1 [file cancers-14-04311-s001.zip › Supplementary Table S1 Antibodies Used for Mass Cytometry.pdf]

Table S1 Antibodies Used for Mass Cytometry

Medal	Antigen	Medal	Antigen
^{197}Au	CD4	^{142}Nd	TCRgd
^{209}Bi	CD45RA	^{143}Nd	CD184 (CXCR4)
^{161}Dy	CD152 (CTLA4)	^{144}Nd	CD134 (OX40)
^{162}Dy	Foxp3	^{145}Nd	CD103 (ITGAE)
^{163}Dy	CD137 (4-1BB)	^{146}Nd	CD49d (ITGA4)
^{164}Dy	RORγt	^{148}Nd	CD185 (CXCR5)
^{166}Er	perforin	^{150}Nd	RUNX3
^{167}Er	KLRG1	^{141}Pr	CD56
^{168}Er	T-bet	^{198}Pt	CD8a
^{170}Er	CD366 (Tim-3)	^{147}Sm	CD197 (CCR7)
^{151}Eu	CD45RO	^{149}Sm	CD279 (PD-1)
^{153}Eu	CD69	^{152}Sm	CD223 (LAG3)
^{155}Gd	CD49a (ITGA1)	^{154}Sm	TIGIT
^{156}Gd	Hobit	^{159}Tb	CD357 (GITR)
^{157}Gd	CD183 (CXCR3)	^{169}Tm	Ki67
^{158}Gd	BCL6	^{89}Y	CD45
^{160}Gd	CD186 (CXCR6)	^{171}Yb	GATA-3
^{165}Ho	CD278 (ICOS)	^{172}Yb	Eomes
^{115}In	CD3	^{173}Yb	Granzyme B
^{139}La	CD44	^{174}Yb	CD252 (OX40L)
^{175}Lu	CD16	^{176}Yb	Blimp-1
