# Supplementary figures and images for: Single-Cell Profiling of the Immune Atlas of Tumor-Infiltrating Lymphocytes in Endometrial Carcinoma
Source: Cancers (Basel). 2022 Sep 2;14(17):4311. doi: 10.3390/cancers14174311 (PMC9455014; doi:10.3390/cancers14174311)

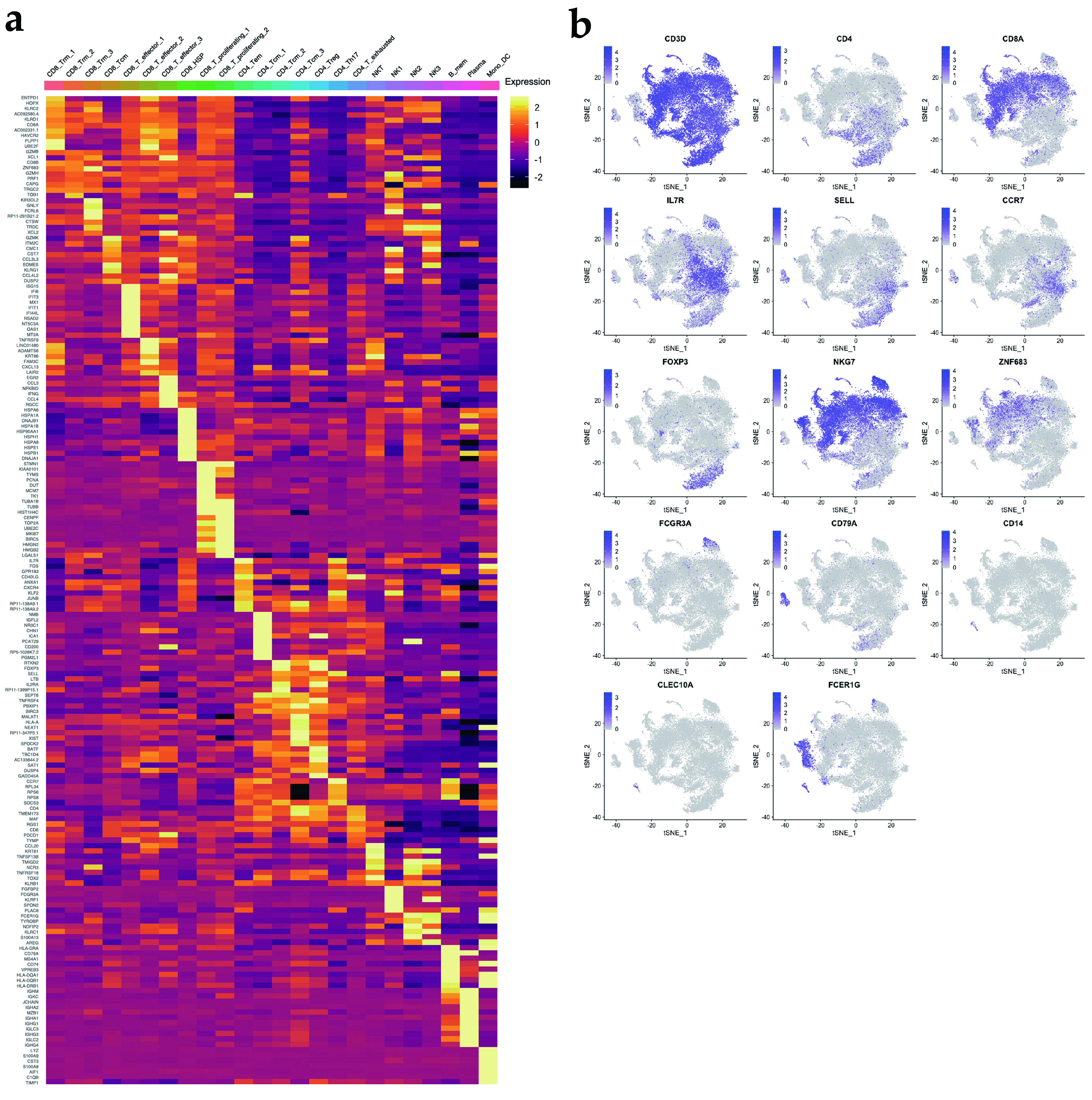

Supplement: Supplementary file 1 [file cancers-14-04311-s001.zip › Fig S1.jpg]

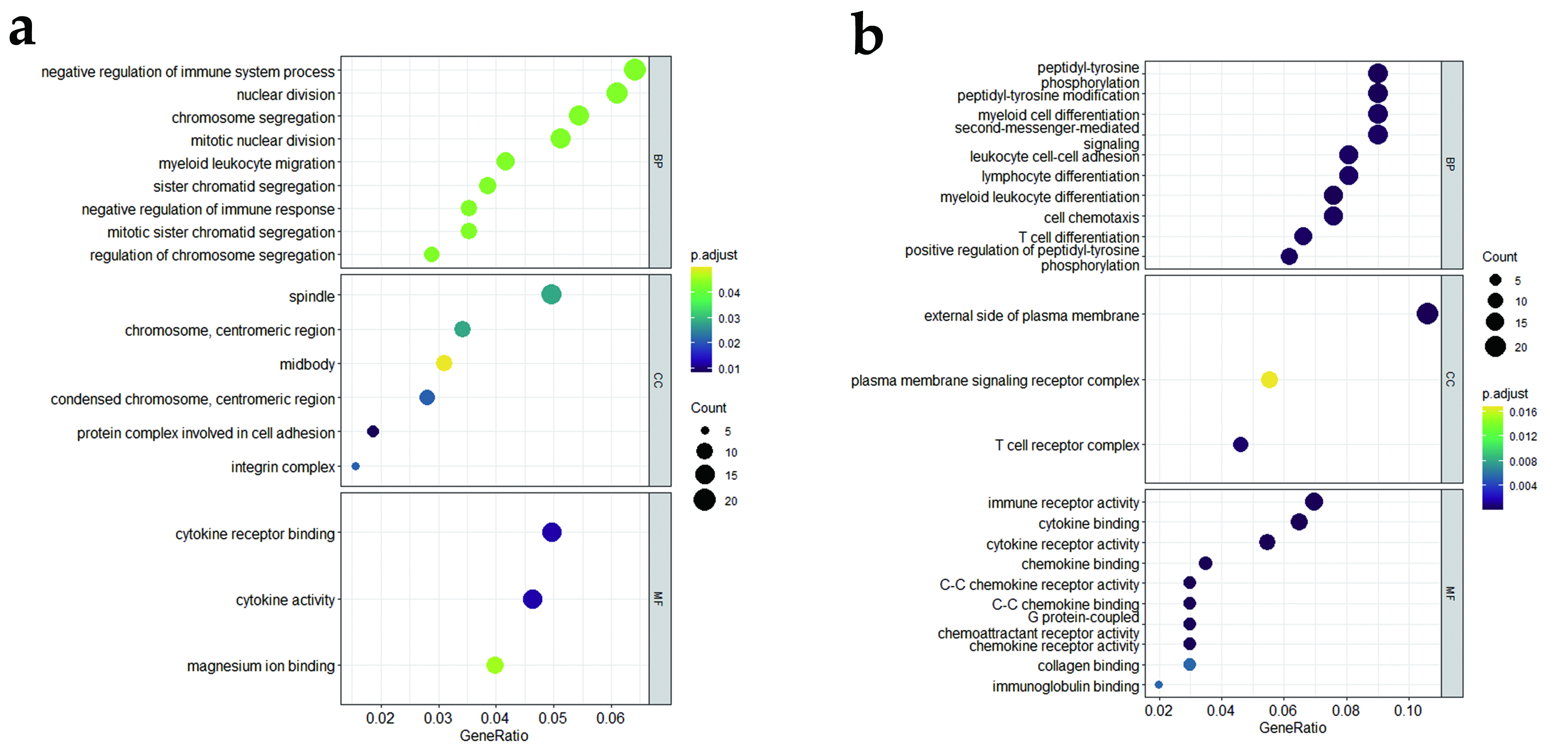

Supplement: Supplementary file 1 [file cancers-14-04311-s001.zip › Fig S2.jpg]

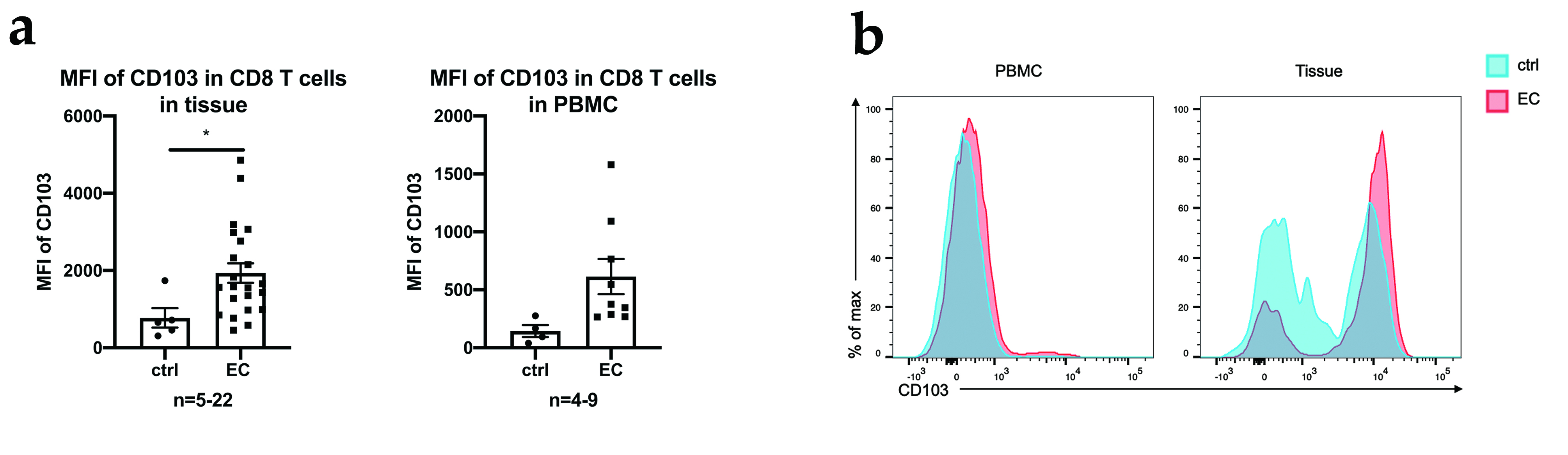

Supplement: Supplementary file 1 [file cancers-14-04311-s001.zip › Fig S3.jpg]
